# Apremilast ameliorates ox-LDL-induced endothelial dysfunction mediated by KLF6

**DOI:** 10.18632/aging.103665

**Published:** 2020-10-14

**Authors:** Hao Wang, Guang Yang, Qian Zhang, Xiao Liang, Yang Liu, Meng Gao, Yutao Guo, Li Chen

**Affiliations:** 1 Department of Cardiology, The Second Medical Center, National Clinical Research Center for Geriatric Diseases, Chinese PLA General Hospital, Beijing 100853, China; 2 Department of Nephrology, The Second Medical Center, National Clinical Research Center for Geriatric Diseases, Chinese PLA General Hospital, Beijing 100853, China; 3 Department of Endocrinology, The Seventh Medical Center, Chinese PLA General Hospital, Beijing 100700, China; 4 Department of Cardiology, The First Medical Center, Chinese PLA General Hospital, Beijing 100853, China; 5 Department of General Practice, The First Medical Center, Chinese PLA General Hospital, Beijing 100853, China

**Keywords:** atherosclerosis, ox-LDL, endothelial dysfunction, apremilast, KLF6

## Abstract

Apremilast is a phosphodiesterase 4 (PDE4) inhibitor used in the treatment of psoriasis and several other inflammatory diseases. Interest has been expressed in seeking out therapies that address both psoriasis and atherosclerosis. In the present study, we explored the effects of apremilast in human aortic endothelial cells (HAECs) exposed to oxidized low-density lipoprotein (ox-LDL) to simulate the atherosclerotic microenvironment *in vitro*. Our findings indicate that apremilast may reduce the expression of lectin-like oxidized-low-density-lipoprotein receptor-1 (LOX-1), the main ox-LDL scavenging receptor. Apremilast also inhibited the expression of tumor necrosis factor-α (TNF-α), interleukin-6 (IL-6), and interleukin-8 (IL-8), which are deeply involved in the chronic inflammatory response associated with atherosclerosis. Interestingly, we found that apremilast inhibited the attachment of U937 monocytes to HAECs by reducing the expression of the chemokine monocyte chemotactic protein 1 (MCP-1) and the cellular adhesion molecule vascular cell adhesion molecule-1 (VCAM-1). This effect was found to be mediated through the rescue of Krüppel like factor 6 (KLF6) expression, which was reduced in response to ox-LDL via increased phosphorylation of c-Jun N-terminal kinase (JNK). These findings suggest a potential role for apremilast in the treatment of atherosclerosis.

## INTRODUCTION

Atherosclerotic cardiovascular disease (CVD) is considered the global leading cause of death. Atherosclerosis is a chronic inflammatory disease characterized by the slow accumulation of lipids and inflammatory immune cells within the arterial wall. While asymptomatic in the early stages, progressive hardening and thickening of the arterial walls leads to arterial occlusion, while the erosion or rupture of atherosclerotic plaques can induce thrombus formation, both of which result in ischemic sequelae, such as myocardial infarction or stroke [1]. Oxidized low-density lipoprotein (ox-LDL) refers to native LDL cholesterol that has undergone oxidation by reactive oxygen species (ROS). Elevated ox-LDL is widely regarded as a major risk factor for atherosclerosis. The lectin-like ox-LDL receptor-1 (LOX-1) is activated by ox-LDL and mediates cellular injury in atherosclerosis. Blocking LOX-1 expression is regarded as a method for reducing the disease burden of atherosclerosis [2]. ROS play a critical role in normal physiology by regulating the inflammatory response, cellular growth and apoptosis, and vascular tone. However, overproduction of ROS leads to a state of oxidative stress and endothelial cell dysfunction [3].

Endothelial cells regulate vascular tone, blood pressure, perfusion, and the aggregation and adhesion of immune cells and platelets. Endothelial dysfunction is considered a predictor of CVD and mortality [4, 5]. The production of chemokines and adhesion molecules, such as monocyte chemoattractant protein-1 (MCP-1) and vascular cellular adhesion molecule-1 (VCAM-1), attracts monocytes to attach to the endothelium. These immune cells then infiltrate the arterial wall where they become macrophages that scavenge ox-LDL to become inflammatory foam cells [6–8]. The expression of pro-inflammatory cytokines, such as tumor necrosis factor-α (TNF-α), interleukin-6 (IL-6), and IL-8, further drives endothelial damage and disease progression [9–10]. Krüppel-like factor 6 (KLF6) is a zinc-finger transcription factor that has been recognized as a critical regulator of vascular remodeling and angiogenesis. KLF6 mediates cellular differentiation, proliferation, apoptosis, and angiogenesis by acting as a damage-response factor and translocating to the nucleus in response to injury, where it mediates the activation of a wide range of genes by binding to their promoters via interaction with Sp1. These include plasminogen activator, endoglin, collagen α1 (I), transforming growth factor-β1 (TGF-β1), and TGF-β receptor type I [11–13]. KLF6 expression is increased in response to ox-LDL exposure and has been shown to guide macrophages toward the anti-inflammatory M1 phenotype [14, 15]. Modulating the expression of KLFs including KLF6 is considered a potential treatment strategy for atherosclerosis.

Apremilast is a cAMP phosphodiesterase-4 (PDE4) inhibitor used for the treatment of psoriasis and other inflammatory diseases. PDE4 has been well documented as a regulator of inflammation and inhibition of PDE4 has been demonstrated to exert anti-inflammatory effects in diseases including asthma, lung neutrophilia, arthritis, inflammatory bowel disease, multiple sclerosis, osteoporosis, and others [16]. Additionally, apremilast has sought approval for the treatment of ankylosing spondylitis, Behçet’s syndrome, atopic dermatitis, and rheumatoid arthritis [17]. Interestingly, apremilast has also been shown to reduce foam cell formation and increase HDL-mediated cholesterol efflux [18]. However, the mechanism of apremilast in atherosclerosis remains to be fully elucidated. In the present study, we investigated the effects of apremilast in human aortic endothelial cells (HAECs) challenged with ox-LDL to simulate the atherosclerotic endothelium *in vitro*. Our findings indicate that apremilast reduced the expression of inflammatory cytokines, chemokines, adhesion molecules, and the LOX-1 receptor. Interestingly, these effects were mediated through c-Jun N-terminal kinase (JNK)/KLF6 signaling.

## RESULTS

### Expression of LOX-1

**Figure 1:**
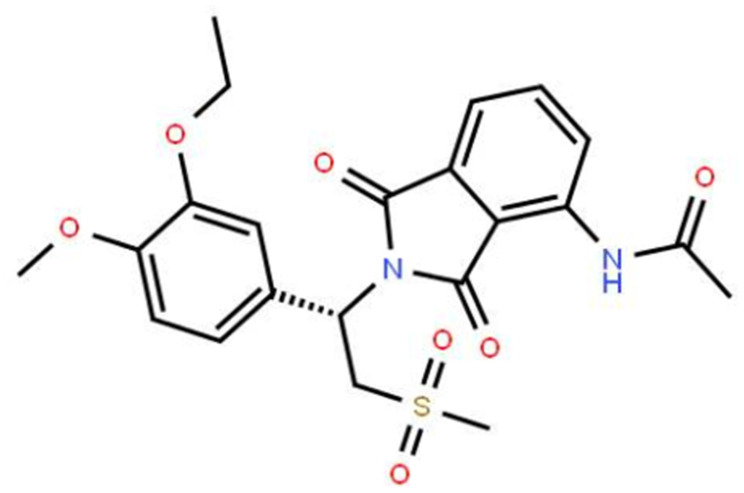
**Molecular structure of apremilast.**

**Figure 2:**
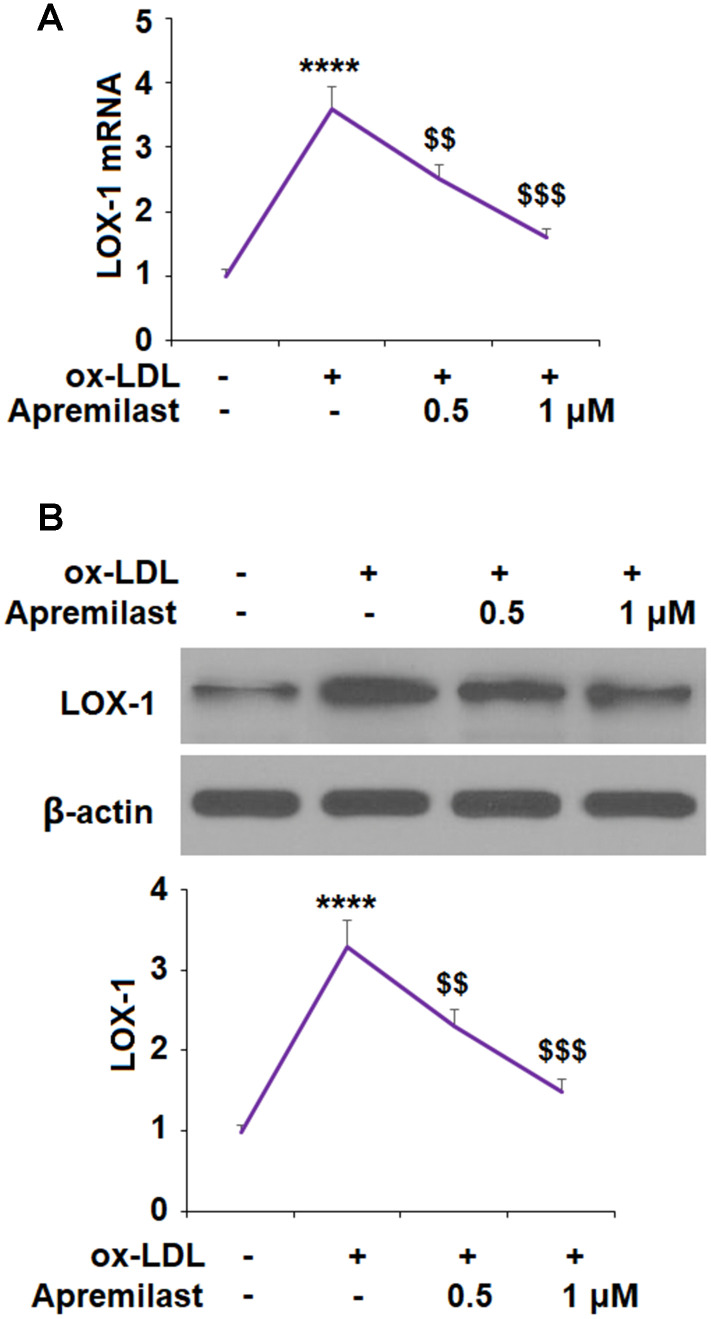
**Apremilast reduced ox-LDL-induced expression of LOX-1 in HUVECs.** Cells were treated with ox-LDL (100 μg/mL) in the presence or absence of apremilast (0.5, 1 μM) for 24 h. (**A**) mRNA of LOX-1; (**B**) Protein of LOX-1 (****, P<0.0001 vs. non-treatment group; $$, $$$, P<0.01, 0.001 vs. ox-LDL group, n=4-5).

The molecular formula of apremilast is shown in Figure 1. It is a small molecule inhibitor weighing 460.5 g/mol with the molecular formula C_22_H_24_N_2_O_7_S (PubChem, 2020) We began by measuring the effect of apremilast on the expression of LOX-1 induced by ox-LDL. The results of real-time PCR and western blot analysis in Figure 2 show that apremilast reduced the expression and protein secretion of LOX-1 in a dose-dependent manner. While ox-LDL increased the mRNA expression of LOX-1 to 3.6-fold, it was reduced to 2.5- and 1.6-fold by 0.5 and 1 μM apremilast, respectively. A similar effect was observed at the protein level.

### Expression of proinflammatory cytokines

**Figure 3:**
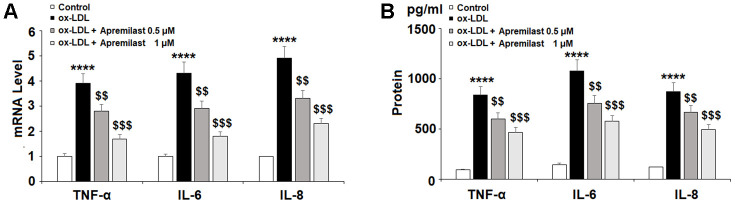
**Apremilast ameliorated ox-LDL-induced expression and secretion of pro-inflammatory cytokines in HUVECs.** Cells were treated with ox-LDL (100 μg/mL) in the presence or absence of apremilast (0.5, 1 μM) for 24 h. (**A**) mRNA of TNF-α, IL-6, and IL-8; (**B**) Secretions of TNF-α, IL-6, and IL-8 (****, P<0.0001 vs. non-treatment group; $$, $$$, P<0.01, 0.001 vs. ox-LDL group, n=4-5).

Next, we set out to establish an anti-inflammatory profile of apremilast in HAECs. Upon exposure to 100 μg/ml ox-LDL, the mRNA levels of TNF-α, IL-6, and IL-8 increased 3.9-, 4.3-, and 4.9-fold. Meanwhile, treatment with apremilast dose-dependently reduced the expression of these cytokines, falling to 1.7-, 1.8-, and 2.3-fold, respectively, in the presence of 1 μM apremilast. Similarly, the significant increase in protein expression of TNF-α, IL-6, and IL-8 was dose-dependently reduced by apremilast treatment (Figure 3B). Therefore, apremilast displays a robust anti-inflammatory effect in ox-LDL-induced HAECs.

### Adhesion of monocytes to endothelial cells

**Figure 4:**
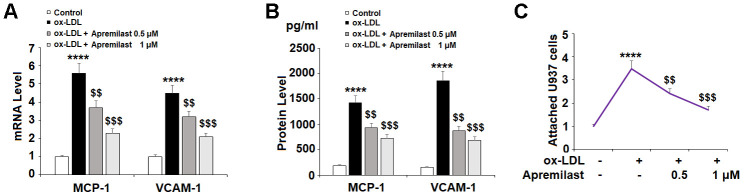
**Apremilast prevented ox-LDL-induced expression of MCP-1 and VCAM-1 and attenuated ox-LDL-induced attachment of U937 monocytes to HUVECs.** Cells were treated with ox-LDL (100 μg/mL) in the presence or absence of apremilast (0.5, 1 μM) for 24 h. (**A**) mRNA of MCP-1 and VCAM-1; (**B**) Protein of MCP-1 and VCAM-1; (**C**) Attachment of U937 monocytes to HUVECs was measured by calcein-AM staining (****, P<0.0001 vs. non-treatment group; $$, $$$, P<0.01, 0.001 vs. ox-LDL group, n=4-5).

The recruitment and adhesion of monocytes to endothelial cells is an important causative event in the development of atherosclerotic plaques. To determine whether apremilast can hinder this process, we measured its effects on the levels of the chemokine MCP-1 and the adhesion molecule VCAM-1. In the presence of ox-LDL alone, the expression of MCP-1 and VCAM-1 was significantly increased at both the mRNA and protein levels. However, treatment with the two doses of apremilast exerted a distinct suppressive effect (Figure 4A and 4B).

We confirmed that the suppression of MCP-1 and VCAM-1 reduced the adhesion of monocytes to endothelial cells by performing a cellular adhesion assay using HAECs and U937 monocytes stained with calcein-AM. The results in Figure 4C show that apremilast treatment did indeed inhibit ox-LDL-induced monocyte attachment to endothelial cells, demonstrating a potentially valuable anti-atherosclerotic effect.

### Involvement of the KLF6 pathway

**Figure 5:**
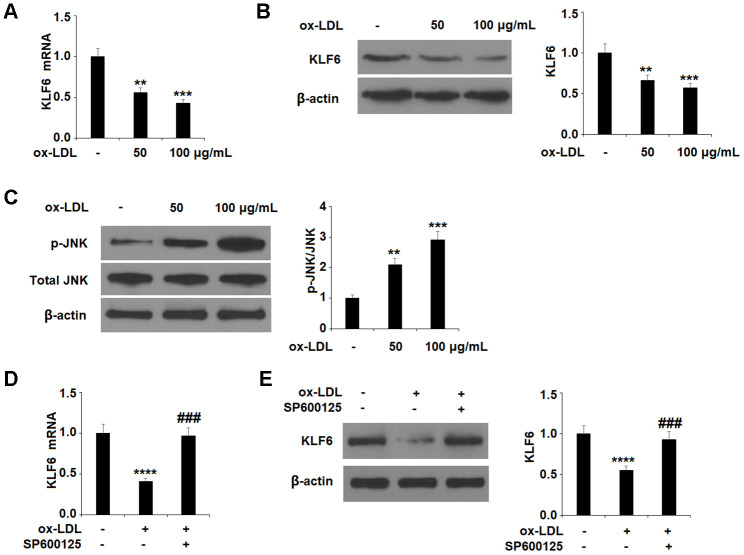
**Ox-LDL reduced the expression of KLF6 mediated by JNK in HUVECs.** (**A**, **B**) Cells were treated with ox-LDL (50, 100 μg/mL) for 24 h. mRNA and protein of KLF6 were measured by real-time PCR and western blot analysis respectively. (**C**) Cells were treated with ox-LDL (50, 100 μg/mL) for 2 h. Phosphorylated JNK was measured by western blot analysis. (**D**, **E**) Cells were treated with ox-LDL (100 μg/mL) in the presence of the JNK inhibitor SP600125 (10 μM) for 24 h. mRNA and protein of KLF6 were measured, respectively (**, ***, ****, P<0.01, 0.001, 0.0001 vs. non-treatment group;, ###, P<0.0001 vs. ox-LDL treatment group, n=4-5).

Finally, we explored the involvement of the KLF6 pathway through a series of experiments. First, we determined that ox-LDL dose-dependently downregulates the expression of KLF6 (Figure 5A, 5B), indicating a possible role of KLF6 in mediating ox-LDL-induced injury. Next, we determined that ox-LDL dose-dependently increased the phosphorylation of JNK (Figure 5C).

**Figure 6:**
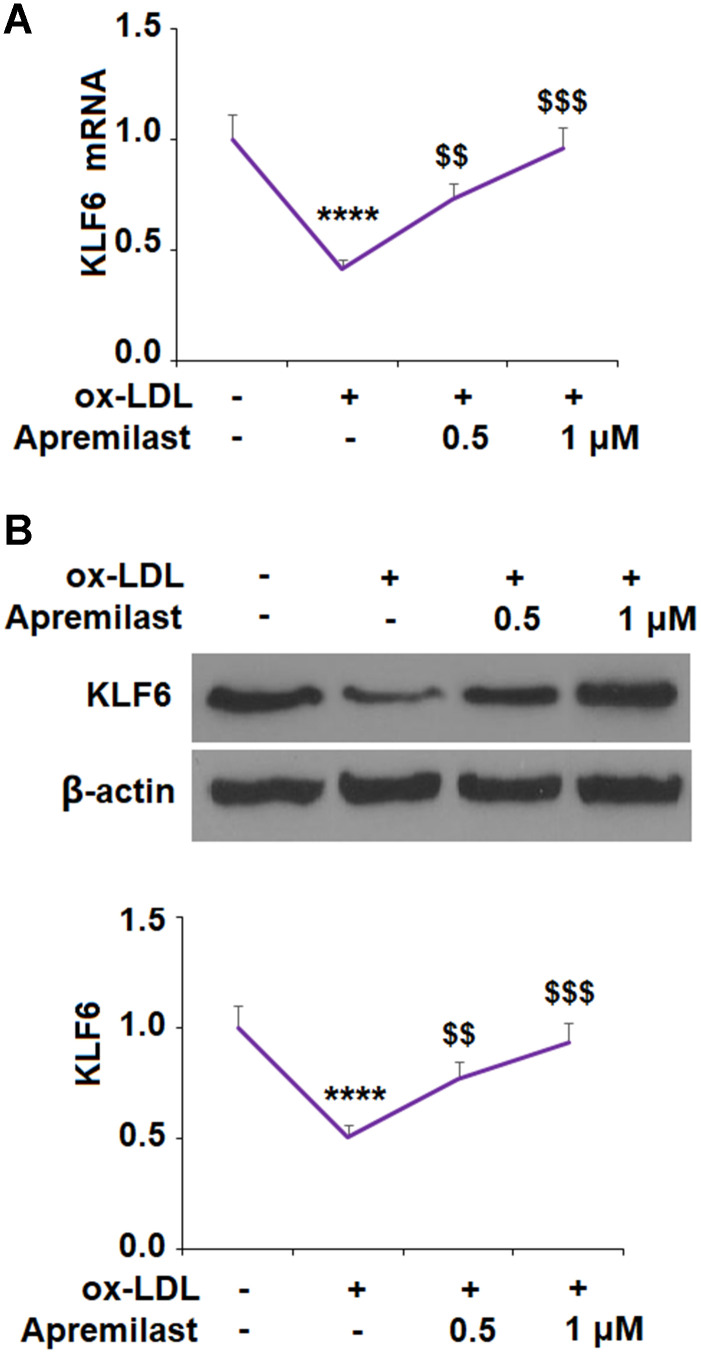
**Apremilast ameliorated ox-LDL-induced reduction of KLF6 in human umbilical vein endothelial cells (HUVECs).** Cells were treated with ox-LDL (100 μg/mL) in the presence or absence of apremilast (0.5, 1 μM) for 24 h. (**A**) mRNA of KLF6; (**B**) Protein of KLF6 (*, #, $, P<0.01 vs. previous column group, n=4-5).

To ensure that JNK phosphorylation was involved in the ox-LDL-mediated decrease in KLF6 expression, we employed the JNK inhibitor SP600125. The results in Figure 5D, 5E confirm that JNK is required for ox-LDL-mediated reduction of KLF6 expression. Next, we measured the effects of apremilast on ox-LDL-induced inhibition of KLF6. As shown in Figure 6A and 6B, treatment with the two doses of apremilast remarkably rescued the mRNA and protein expression of KLF6 to near basal levels in the presence of ox-LDL.

**Figure 7:**
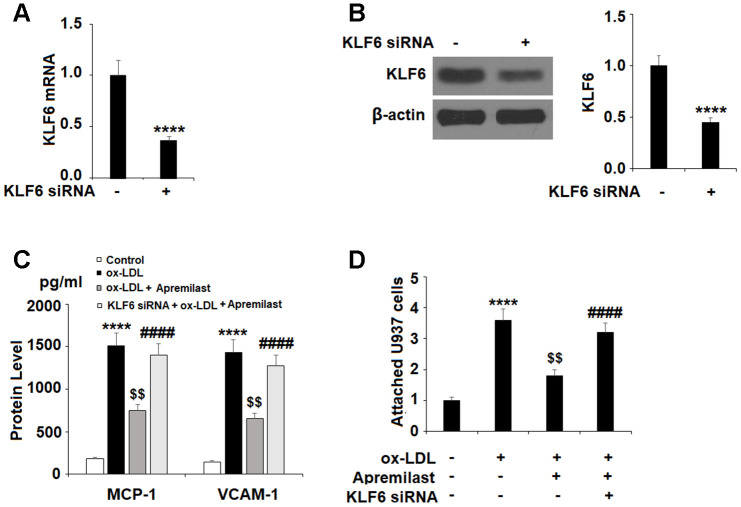
**The protective effects of apremilast against ox-LDL-induced attachment of monocytes to HUVECs were mediated by KLF6.** HUVECs were transfected with KLF6 siRNA. (**A**, **B**) Real time PCR and western blot analysis revealed successful knockdown of KLF6; (**C**) Protein of MCP-1 and VCAM-1; (**D**) Attachment of U937 monocytes to HUVECs (****, P<0.0001 vs. non-treatment group; $$, P<0.01 vs. ox-LDL treatment group; ####, P<0.0001 vs. ox-LDL+ group, n=4-5).

Lastly, we tested whether the effects of apremilast on MCP-1 and VCAM-1 expression were mediated through KLF6 by performing a KLF6 siRNA knockdown. Successful knockdown of KLF6 is shown in Figure 7A and 7B. As shown in Figure 7C, knockdown of KLF6 abolished the apremilast-mediated suppression of MCP-1 and VCAM-1. Concordantly, the results in Figure 7D show that knockdown of KLF6 also abolished the inhibition of monocyte attachment to endothelial cells induced by apremilast.

## DISCUSSION

As one of the leading causes of death, treatments to prevent or halt the progression of atherosclerosis are of considerable value. In the present study, we investigated the effects of the PDE4 inhibitor apremilast *in vitro* in the context of atherosclerosis using an ox-LDL-challenged HAEC model. Apremilast was originally developed as a treatment for psoriasis and psoriatic arthritis. Its main mechanism of action is inhibition of PDE4 enzymes, which hydrolyze cyclic adenosine monophosphate (cAMP). cAMP is a second messenger protein involved in the regulation of intracellular proinflammatory and anti-inflammatory pathway signaling [19]. Interestingly, psoriasis and atherosclerosis have been revealed to share common pathogenic and inflammatory mechanisms. For example, endothelial invasion by inflammatory immune cells occurs in both diseases. Thus, treatments that address both psoriasis and atherosclerosis are considered to be valuable [20]. The findings of the present study indicate that apremilast may be just such a treatment.

LOX-1 is the main scavenging receptor for ox-LDL. Enhanced expression of LOX-1 leads to increased foam cell formation, as LOX-1-expressing macrophages in the arterial wall display increased uptake of ox-LDL [21]. Targeting LOX-1 is considered an attractive treatment approach for atherosclerosis [22]. Here, we found that apremilast significantly decreased the expression of LOX-1, suggesting an inhibitory effect against foam cell formation. Proinflammatory cytokines are a major effector of atherogenesis. Inhibition of PDE4 by drugs including apremilast is known to suppress the expression of TNF-α. Apremilast has been shown to inhibit the expression of these three cytokines. For example, a phase III randomized clinical trial of apremilast found a significant decrease in TNF-α, IL-6, and IL-8 levels in psoriatic arthritis patients after 24 weeks of BID dosing [23]. Meanwhile, apreimlast-mediated inhibition of IL-8 was found to reduce neutrophil chemotaxis by 75% as well as inhibit the adhesion of neutrophils to human umbilical vein endothelial cells [24]. Another PDE4 inhibitor, NCS613 has been shown to reduce TNF-α, IL-6, and IL-8 expression via inhibition of p38 mitogen-activated protein kinase (MAPK) and nuclear factor-κB (NF-κB) [25]. Here, we found that apremilast significantly decreased ox-LDL-induced TNF-α, IL-6, and IL-8 expression, however, whether these pathways were involved remains unclear.

The adhesion of monocytes to endothelial cells is a major event in the pathogenesis of atherosclerosis. MCP-1 and VCAM-1 play a pivotal role in recruiting monocytes to the endothelium and triggering their rolling and adhesion. Apremilast has been shown to inhibit MCP-1 expression in dermal myofibroblasts and human primary peripheral blood mononuclear cells [26, 16]. Previous research on PDE4 inhibitors has demonstrated a suppressive effect on the expression of VCAM-1 as well as intercellular adhesion molecule-1 (ICAM-1), another important adhesion molecule [27]. However, there is limited research regarding the influence of apremilast on adhesion molecule activity. Here, we found that apremilast reduced the expression of both MCP-1 and VCAM-1 induced by ox-LDL, which resulted in reduced adhesion of U937 monocytes to HAECs. Importantly, we found that this inhibition of monocyte adhesion was mediated through the KLF6 signaling pathway. KLF6 is mainly expressed in endothelial cells and has been shown to mediate macrophage phenotype polarization, which plays a major role in the pathogenesis and regression of atherosclerosis [28, 29]. Here, we found that ox-LDL suppressed KLF6 activity through reducing the phosphorylation of JNK. Previous research has shown that apremilast can reduce JNK expression in cardiac tissue [30]. Additionally, our results show that apremilast-mediated rescue of KLF6 expression prevented the attachment of monocytes to endothelial cells via reduction of MCP-1 and VCAM-1, which was abolished in KLF6-silent cells. Thus, apremilast may prevent the progression of atherosclerosis via several mechanisms, including the inhibition of ox-LDL uptake via reduced LOX-1 expression, suppression of the inflammatory response via reduced TNF-α, IL-6, and IL-8 expression, and reduced adhesion of monocytes to endothelial cells via KLF6-mediated suppression of MCP-1 and VCAM-1 expression.

There are several limitations to this study. Firstly, an *in vitro* assay was used for this preliminary assessment of apremilast in atherosclerosis. Future studies will include a more ideal animal model so that we may observe whether these findings hold true *in vivo*. Secondly, previous research has demonstrated the involvement of the NF-κB and other pathways in mediating the anti-inflammatory effects of apremilast [25]. It is unclear whether these signaling pathways are involved in the effects of apremilast observed herein. Finally, although likely, it remains unclear whether apremilast rescued the expression of KLF6 through the inhibition of JNK. Further studies will help to elucidate the exact mechanisms involved in the novel atheroprotective effects of apremilast described in the present study.

## MATERIALS AND METHODS

### Cell culture and treatment

Primary human aortic endothelial cells (HAECs) and U937 monocytic cell line were obtained from the American Type Culture Collection (ATCC PCS-100-011, Rockville, MD, USA). HAECs were cultured at 37 °C in a 5% (v/v) CO_2_/95% (v/v) nitrogen atmosphere in low passage numbers (<5) in 2% serum endothelial growth media (EGM2) (Lonza, Switzerland) supplemented with fetal bovine serum (5%, Invitrogen, Carlsbad, CA, USA) and 1% antibiotics (penicillin/streptomycin) (Invitrogen) as previously described [31]. U937 cells used in the cellular adhesion assay were grown in 10% fetal serum containing in Dulbecco's Modified Eagle Medium (DMEM) (Thermo Fisher Scientific, USA) in a 5% (v/v) CO_2_/95% (v/v) nitrogen incubator at 37 °C. For the KLF6 knockdown experiment, HAECs were transiently transfected with KLF6 siRNA using Lipofectamine 3000 (Thermo Fisher Scientific, USA) in accordance with the manufacturer’s instructions. For all experiments, HAECs were stimulated with 100 mg/L ox-LDL in the presence or absence of apremilast (Celgene Corporation, NJ, USA) (0.5 or 1 μM) for 24 h.

### Real-time PCR

To determine the expression of the target genes, RNA was extracted from HAECs using an RNeasy Micro Kit (Cat.74004, Qiagen, USA) in accordance with the manufacturer’s instructions. RNA concentrations were quantified using a Nanodrop spectrophotometer. Then, RT-qPCR (Invitrogen, USA) was performed by synthesizing cDNA from 1 μg isolated RNA using iScript™ Reverse Transcription Supermix (Bio-Rad, #1708840, USA). SYBR-based real-time PCR was performed to detect the total mRNA transcripts of human LOX-1, TNF-α, IL-6, IL-8, MCP-1, VCAM-1, and KLF6 on an ABI 7500 platform [32]. Primer sequences have been listed in Table 1.

**Table 1:** Primer sequences.

**Target gene**	**Upstream sequence (5’-3’)**	**Downstream sequence (5’-3’)**
IL-6	5’- TTGGGAAGGTTACATCAGATC-3’;	5’- GGGTTGGTCCATGTCAATTT -3’;
IL-8	5’-ATGACTTCCAAGCTGGCCGTGGCT-3’;	5’-TCTCAGCCCTCTTCAAAAACTTCTC -3’;
TNF-α	5’-CCAGACCCTCACACTCAGATC-3’;	5’- CACTTGGTGGTTTGCTACGAC -3’;
MCP-1	5’- ATGCAATCAATGCCCCAGTC-3’;	5’- TGCAGATTCTTGGGTTGTGG-3’;
VCAM-1	TCTC 5’- CTTAAAATGCCTGGGAAGATGGT-3’;	5’ - GTCAATGAGACGGAGTCACCAAT-3’;
KLF6	5’-GTGACAAGGGTAATGGCGAC-3’;	5’-ATGAGCATCTGTAAGGCTTTTCT-3’;
LOX-1	5’-TTACTCTCCATGGTGGTGGTGCC-3’;	5’-AGCTTCTTCTGCTTGTTGCC-3’;
GAPDH	5’- GGAGCGAGATCCCTCCAAAAT-3’;	5’- GGCTGTTGTCATACTTCTCATGG-3’.

### Western blot analysis

After the indicated treatment, HAECs were lysed using radioimmunoprecipitation assay (RIPA) buffer (Sigma-Aldrich, USA) containing protease and phosphatase inhibitors (Sigma-Aldrich, USA). Cytoplasm was removed using hypotonic buffer to obtain the nuclear extracts via lysis. Then, 20 μg cell lysates was immobilized using 10% sodium dodecyl sulfate polyacrylamide gel electrophoresis (SDS-PAGE). The protein mix was then transferred onto polyvinylidene fluoride (PVDF) membranes (Bio-Rad, USA) to separate the proteins according to size. The membranes were then blotted against their specific antibodies and corresponding secondary antibodies. The immunoblot bands were visualized using Pierce™ ECL Plus western blotting substrate (Catalog # 32132) [33]. The following antibodies were used: p-JNK (1:1000, # 9255, Cell Signaling Technology, USA); JNK (1:3000, #9252, Cell Signaling Technology, USA); LOX-1 (1:2000, ab214427, Abcam); KLF6 (1:1000, #AF3499, R&D Systems); β-actin (1:5000, #4970, Cell signaling technology, USA); HRP-linked anti-rabbit IgG antibody (1:2000, #7074, Cell signaling technology, USA); HRP-linked anti-mouse IgG antibody (1:2000, #7076, Cell Signaling Technology, USA).

### ELISA

Enzyme-linked immunosorbent assay (ELISA) was used to detect the protein secretion of the target genes. Specific ELISA kits were purchased from R&D Systems and used in accordance with the manufacturer’s instructions. The kits used in this study were: human TNF-α Quantikine ELISA Kit (#DTA00C, R&D Systems), human IL-6 Quantikine ELISA Kit (#D6050, R&D Systems), human CCL2/MCP-1 Quantikine ELISA Kit (#DCP00, R&D Systems), human VCAM-1 DuoSet ELISA Kit (#DY809, R&D Systems). Briefly, 50 μL HAEC culture media was collected and added into ELISA plates. After incubation for 2 h at room temperature, 96-well ELISA plates were washed for 4 times and incubated with 100 μL of diluted detection antibody for another 1 h. After incubation with secondary antibodies for 30 minutes at room temperature, 100 μL substrate was added and incubated for 30 minutes at room temperature. 100 μL of stop solution was used to stop the reaction. The absorbance at 450 nm was measured.

### Cellular adhesion assay

The cell-permanent dye calcein a^c^etoxymethyl ester (calcein-AM) was used to quantify the attachment of monocytes to endothelial cells. HAECs were grown to full confluence in 6-well plates exposed to the indicated treatment. U937 monocytes were seeded into 6-well plates at a density of 2 × 10^5^ and labeled with calcein-AM (Invitrogen, USA) for 30 min. The labeled monocytes were then incubated with HAECs for 2 h. The wells were rinsed 3 times with PBS buffer containing 1% BSA to remove the free-floating cells. The plates were fixed with 4% paraformaldehyde, and a fluorescence microscope (Excitation wavelength/Emission wavelength: 490 nm/515 nm) was used to visualize the calcein-labeled U937 monocytes attached to HAECs.

### Knockdown of KLF6

HAECs were loaded into 6-well plates and incubated in antibiotic-free EBM-2 medium 1 day prior to transfection. Then, KLF6-specific siRNA was transfected into HAECs by incubating with Lipofectamine 2000 (Invitrogen, USA) at room temperature for 30 min. At 3 h post-transfection, the medium was replaced with EBM-2 and the cells were cultured for an additional 48 hours as previously described [34].

### Statistical analysis

Experiments were repeated for 3 times. The statistical significance of differences was assessed using one-way analysis of variance (ANOVA) followed by the Bonferroni post-hoc test using SPSS (Version 19.0). A P value of less than 0.05 was determined to be statistically significant. The data from all experiments are presented as means ± standard deviation (SD).
